# Supplementary material for: Effects of Different Marinades and Types of Grills on Polycyclic Aromatic Hydrocarbon Content in Grilled Chicken Breast Tenderloins
Source: Foods. 2024 Oct 24;13(21):3378. doi: 10.3390/foods13213378 (PMC11545549; doi:10.3390/foods13213378)
Supplement: Supplementary file 1 [file foods-13-03378-s001.zip › foods-3256954-supplementary.pdf]

Table S1. Content of individual PAHs in chicken breast tenderloins in different marinades and unmarinated grilled on the charcoal grill without a tray (W1) and with an aluminum tray (W2).

PAH	PAH content [µg/kg]							
	**KMUW1**	**KMKW1**	**KMMW1**	**KBMW1**	**KMUW2**	**KMKW2**	**KMMW2**	**KBMW2**
Phen	36.42±0.06	50.78±3.37	17.61±0.81	51.80±2.63	23.87±1.35	34.76±3.95	14.47±0.25	18.02±0.33
Anthr	16.61±0.36	7.69±0.75	5.88±0.30	26.04±0.87	6.20±0.07	2.53±0.14	3.40±0.34	4.48±0.10
F	24.11±0.04	28.19±0.32	19.98±0.57	32.63±1.32	18.20±1.09	7.82±0.411	9.74±0.66	16.12±0.56
Pyr	11.99±1.19	6.17±0.47	3.89±0.28	6.48±0.37	2.03±0.07	1.76±0.20	1.93±0.17	2.46±0.20
C[cd]P	nd[x]	nd	nd	nd	nd	nd	nd	nd
B[a]A	1.08±0.03	0.84±0. 05	1.56±0.15	2.17±0.11	0.35±0.02	0.40±0.02	0.55±0.02	0.47±0.01
Chr	0.84±0.02	1.00±0.10	1.03±0.02	1.02±0.06	0.36±0.03	0.44±0.02	0.62±0.06	0.49±0.02
5-MChr	nd	nd	nd	nd	nd	nd	nd	nd
B[j]F	nd	nd	nd	nd	nd	nd	nd	nd
B[b]F	0.72±0.00	0.67±0.02	0.90±0.01	0.71±0.03	0.31±0.02	0.39±0.01	0.58±0.03	0.48±0.02
B[k]F	0.70±0.01	0.58±0.02	0.86±0.02	0.55±0.01	0.32±0.02	0.37±0.02	0.55±0.02	0.45±0.01
B[a]P	1.06±0.03	1.08±0.04	1.52±0.02	1.28±0.02	0.51±0.06	0.66±0.02	0.93±0.03	0.76±0.01
D[ah]A	nd	nd	nd	nd	nd	nd	nd	nd
D[al]P	0.84±0.02	0.75±0.01	0.90±0.03	0.64±0.01	0.49±0.02	0.50±0.03	0.66±0.01	0.60±0.03
B[ghi]P	1.17±0.11	0.85±0.10	1.25±0.17	1.06±0.12	0.70±0.09	0.57±0.09	0.91±0.17	0.65±0.05
I[cd]P	0.62±0.08	0.55±0.02	0.80±0.04	0.47±0.02	0.26±0.03	0.36±0.03	0.49±0.04	0.41±0.03
D[ae]P	nd	nd	nd	nd	nd	nd	nd	nd
D[ai]P	nd	nd	nd	nd	nd	nd	nd	nd
D[ah]P	nd	nd	nd	nd	nd	nd	nd	nd

n = 6 (six samples of every kind of product were analyzed). [x] nd – not detected. K – Chicken breast tenderloins. MU – Universal marinade, MK – Chicken marinade, MM – Honey–mustard marinade, BM – Without marinade. W1 – Charcoal grill without a tray, W2 – Charcoal grill with an aluminum tray, E1 – Ceramic contact grill, E2 – Cast iron contact grill.

Table S2. Content of individual PAHs in chicken breast tenderloins in different marinades and unmarinated grilled on the electric ceramic contact grill (E1) and cast iron contact grill (E2).

PAH	PAH content [μg/kg]							
	KMUE1	KMKE1	KMME1	KBME1	KMUE2	KMKE2	KMME2	KBME2
Phen	6.89±0.35	7.65±1.29	7.21±0.12	5.47±0.17	6.46±0.40	8.18±1.50	7.10±0.01	5.56±0.31
Anthr	1.17±0.09	2.94±0.07	0.91±0.01	1.34±0.04	1.85±0.05	1.27±0.07	0.95±0.03	1.43±0.06
F	1.58±0.16	7.97±0.14	2.27±0.07	2.38±0.05	3.34±0.10	2.85±0.04	2.64±0.11	3.06±0.51
Pyr	0.31±0.01	1.37±0.06	0.59±0.03	0.49±0.02	0.62±0.07	0.67±0.04	0.62±0.03	0.64±0.02
C[cd]P	nd[x]	nd	nd	nd	nd	nd	nd	nd
B[a]A	nd	0.16±0.01	0.44±0.01	0.36±0.00	0.41±0.02	0.50±0.00	0.53±0.01	0.45±0.02
Chr	nd	0.04±0.00	0.44±0.02	0.37±0.02	0.43±0.02	0.37±0.03	0.46±0.02	0.46±0.02
5-MChr	nd	nd	nd	nd	nd	nd	nd	nd
B[j]F	nd	nd	nd	nd	nd	nd	nd	nd
B[b]F	nd	0.06±0.00	0.36±0.1	0.29±0.01	0.32±0.02	0.32±0.01	0.43±0.01	0.41±0.03
B[k]F	nd	0.02±0.00	0.34±0.01	0.29±0.01	0.28±0.03	0.28±0.03	0.41±0.00	0.40±0.02
B[a]P	nd	0.05±0.00	0.65±0.00	0.55±0.01	0.51±0.05	0.57±0.03	0.75±0.01	0.73±0.04
D[ah]A	nd	nd	nd	nd	nd	nd	nd	nd
D[al]P	nd	0.07±0.00	0.42±0.01	0.32±0.01	0.40±0.00	0.36±0.04	0.46±0.02	0.44±0.05
B[ghi]P	nd	0.30±0.04	0.54±0.05	0.42±0.00	0.27±0.01	0.44±0.03	0.65±0.07	0.70±0.12
I[cd]P	nd	nd	0.36±0.00	0.26±0.03	0.25±0.02	0.34±0.01	0.43±0.05	0.38±0.05
D[ae]P	nd	nd	nd	nd	nd	nd	nd	nd
D[ai]P	nd	nd	nd	nd	nd	nd	nd	nd
D[ah]P	nd	nd	nd	nd	nd	nd	nd	nd

n = 6 (six samples of every kind of product were analyzed). [x] nd – not detected. K – Chicken breast tenderloins. MU – Universal marinade, MK – Chicken marinade, MM – Honey–mustard marinade, BM – Without marinade. W1 – Charcoal grill without a tray, W2 – Charcoal grill with an aluminum tray, E1 – Ceramic contact grill, E2 – Cast iron contact grill.
